# Effectiveness of a practice change intervention in reducing alcohol consumption in pregnant women attending public maternity services

**DOI:** 10.1186/s13011-022-00490-2

**Published:** 2022-08-31

**Authors:** Tracey W. Tsang, Melanie Kingsland, Emma Doherty, John Wiggers, John Attia, Luke Wolfenden, Adrian Dunlop, Belinda Tully, Ian Symonds, Chris Rissel, Christophe Lecathelinais, Elizabeth J. Elliott

**Affiliations:** 1 Discipline of Child and Adolescent Health, Faculty of Medicine and Health, The University of Sydney Children’s Hospital at Westmead Clinical School, Sydney, NSW Australia; 2 Sydney Children’s Hospital Network, Kids Research, Sydney, NSW Australia; 3 Hunter New England Population Health, Hunter New England Local Health District, Longworth Ave, Locked Bag 10, Wallsend, NSW 2287 Australia; 4 School of Medicine and Public Health, The University of Newcastle, Callaghan, NSW Australia; 5 Hunter Medical Research Institute, New Lambton Heights, NSW 2305 Australia; 6 School of Medicine and Public Health, College of Health, Medicine and Wellbeing, The University of Newcastle, Callaghan, NSW Australia; 7 Drug and Alcohol Clinical Services, Hunter New England Local Health District, Newcastle, NSW Australia; 8 The International Medical University, Kuala Lumpur, Malaysia; 9 College of Medicine and Public Health, Flinders University, Casuarina, NT 0909 Australia

**Keywords:** Alcohol Consumption, Pregnancy, Antenatal Care, Australia, Intervention study

## Abstract

**Background:**

The aim of this study was to examine the effect of a practice change intervention to support the implementation of guideline-recommended care for addressing alcohol use in pregnancy on self-reported alcohol use during pregnancy.

**Methods:**

A randomized, stepped-wedge controlled trial in three clusters (sectors) within the Hunter New England Local Health District (NSW, Australia). We evaluated a practice change intervention that supported the introduction of a new model of care for reducing alcohol use in pregnancy, consistent with local and international guidelines, and implemented in random order across the sectors. Each week throughout the study period, pregnant women who attended any public antenatal services within the previous week, for a 27–28 or 35–36 week gestation visit, were randomly sampled and invited to participate in the survey. The intended intervention for all women was Brief advice (to abstain from alcohol and information about potential risks). Women identified as medium-risk alcohol consumers using the Alcohol Use Disorder Identification Test-Consumption (AUDIT-C) were to be offered referral to a phone coaching service, and women identified as high-risk were to be offered referral to a Drug and Alcohol Service. Rates of self-reported alcohol use (AUDIT-C risk level and special occasion drinking) were summarized and compared in groups of women pre-intervention and post-intervention using multivariable logistic regression.

**Results:**

Surveys were completed by 1309 women at pre-intervention and 2540 at post-intervention. The majority of women did not drink during pregnancy (pre-intervention: 89.68%; post-intervention: 90.74%). There was no change in the proportion of women classified as No risk from drinking (AUDIT-C score = 0) or Some risk from drinking (AUDIT-C score ≥ 1) pre- or post-intervention (*p* = 0.08). However, a significant reduction in special occasion drinking was observed (pre-intervention: 11.59%; post-intervention: 8.43%; *p* < 0.001).

**Conclusions:**

Special occasion drinking was reduced following implementation of guideline-recommended care. Failure to change other patterns of alcohol use in pregnancy may reflect barriers to implementing the model of care in antenatal care settings and the need to address other social determinants of alcohol use.

**Trial registration:**

Australian and New Zealand Clinical Trials Registry (registration number: ACTRN12617000882325; date: 16 June 2017).

## Background

With international recognition of the potential harms of prenatal alcohol exposure (PAE), including increased risk of birth defects, neurodevelopmental disorders and FASD [1, 2], guidelines in several countries recommend abstention from alcohol by women who are pregnant or planning a pregnancy [3–5]. Despite consensus that alcohol use in pregnancy causes harm, the estimated global prevalence of PAE is 9.8% with region-based variations, estimates being lowest in the World Health Organization (WHO) Eastern-Mediterranean region and highest in the European region [6]. In Australia, 34.7% to 82.0% of pregnancies are alcohol-exposed [1, 7–9]. Although most women stop drinking alcohol after pregnancy recognition, 18.0% to 25.2% continued to drink throughout pregnancy [1, 10]. Continued alcohol consumption in pregnant Australian women is associated with older age, pre-pregnancy alcohol consumption, alcohol use in a previous pregnancy, and a positive attitude towards alcohol use in pregnancy [10, 11].

The WHO [12] and national clinical guidelines [13] recommend that women receive, as early as possible and throughout pregnancy: i) assessment of alcohol use using a validated tool; ii) advice not to consume alcohol and information about the potential risks to themselves and their baby; and iii) referral to specialist support if required. This is based on systematic review evidence that shows that pregnant women who receive brief psychosocial interventions delivered by healthcare providers are more than twice as likely not to consume alcohol during pregnancy (OR: 2.31; 95% CI: 1.61, 3.32; *p* < 0.001) [14].

Public maternity services are a critical setting for these guideline recommendations addressing alcohol use in pregnancy to be implemented, given the large proportion of pregnant women that attend. Despite this, such care has not been routinely provided to pregnant women in antenatal settings, within Australia and internationally, with low clinician provision of assessment (42%-64%) [15–17], advice (11%-35%) [17, 18] and referral (10%-50%) [17–19].

Two controlled trials have been conducted to evaluate the effectiveness of practice change interventions to support the implementation of clinical guideline recommendations for addressing alcohol consumption during pregnancy in public maternity services. The first, a 2015 trial of action-research and training in four Italian public hospitals, found a significant improvement in health professional knowledge related to alcohol and its use in pregnancy, and in the probability of pregnant women receiving correct advice (intervention: 53% vs control: 20%; RR: 2.66; 95% CI: 1.27, 5.56). However, no significant improvements were observed in the pregnant women’s opinions or attitudes towards alcohol use [20] and the effect of the intervention on women’s alcohol consumption during pregnancy was not reported.

The second was a trial conducted by our group in public maternity services in a single local health district in Australia in 2017–2020 [21]. In this study a multi-strategy practice-change intervention consisting of seven evidence-based implementation strategies increased women’s reported receipt of guideline recommended care elements at three visit types (initial antenatal visit; 27–28 weeks gestation; and 35–36 weeks gestation): assessment of alcohol use using a validated tool (the AUDIT-C) (from 28.4% at Baseline to 40.6% at Follow-up), provision of advice (from 18.7% to 26.7%), complete care relative to the level of alcohol risk (advice and referral) (from 18.5% to 26.6%), and provision of all guideline elements (assessment, advice and referral) (from 12.6% to 19.4%) [22].

Although the intervention was effective in increasing assessment and care provision, its effect on reducing alcohol use in pregnancy has not yet been reported. The aim of the study reported in this paper is to assess the efficacy of the practice change intervention in reducing the proportion of pregnant women consuming alcohol during pregnancy.

## Methods

The protocol has been detailed previously [21]. Relevant details are summarized here. All aspects of the study design, conception through to dissemination was inclusive of Aboriginal peoples to inform cultural inclusion, safety and appropriateness and project governance embedded cultural governance model led by Aboriginal peoples.

### Design and setting

**Fig. 1:**
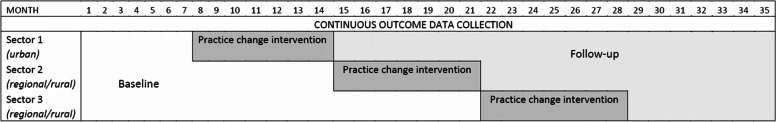
Study timeline for data collection and the practice change intervention [22]

A randomized stepped-wedge controlled trial was conducted in all public maternity services in three sectors within the Hunter New England Local Health District (HNELHD; NSW, Australia) between July 2017 to May 2020. The three sectors comprised one major city (Sector 1: 4300 births/year or 70% of births in the district) and two regional/rural areas (Sector 2: 1200 births/year; Sector 3: 600 births/year). Stepped implementation of the seven-month practice change intervention was initiated in each sector in random order, six months apart. Pre-intervention (baseline) surveys were conducted in the seven-month period preceding the implementation of the practice change intervention at the first sector (with usual practice used as the Control) and Post-intervention (follow-up) surveys occurred up to seven months after the completion of the intervention period in the third sector (Fig. 1) [21]. The randomized stepped-wedge design was chosen for this trial for several reasons including that it provides a similar level of evidence as a parallel cluster randomized controlled trial (RCT); enables the ability to identify secular trends (changes over time) before the intervention is implemented through the sequential implementation of the intervention across three sectors; allows each cluster to act as its own control, thus addressing the practical difficulty of recruiting the number of similar antenatal services required for a parallel cluster RCT; and that it gives all participating services and women the opportunity to receive the intervention [21].

### Randomisation and blinding

Randomisation of the order of intervention delivery to the three sectors was undertaken by an independent statistician (CL) using a computerised random number generator. Randomisation was non-stratified. Study personnel involved in outcome data collection were blinded to the intervention order. Antenatal providers could not be blinded to intervention status due to the nature of the practice change intervention.

### Eligibility and recruitment

#### Maternity services and care providers

The practice change intervention was implemented in all maternity services within the three sectors according to their stepped allocation. Service types included hospital and community-based midwifery services, hospital medical clinics, midwifery continuity of care group practices, Aboriginal maternal and infant health services (AMIHS), and specialist services caring for women with complex pregnancies or social vulnerabilities. Within these services, all antenatal care providers (midwifery and medical staff, and Aboriginal health workers) were eligible to receive the implementation strategies. Clinicians who were not the primary providers of antenatal care were not targeted for the intervention.

#### Pregnant women

All women who attended the participating maternity services had the potential to receive the recommended model of care. During the study period, women were eligible to participate in surveys if they met the following criteria:

- Aged ≥ 18 years
- Were pregnant and between 12–37 weeks gestation
- Had attended a face-to-face antenatal visit in the preceding week for either a visit at 27–28 weeks or 35–36 weeks gestation.

Exclusion criteria were: antenatal care with a private obstetrician; prior selection to participate in the survey within the past four weeks; previously declined participation; had given birth; or had a negative pregnancy outcome (stillbirth or miscarriage) as identified from electronic medical records.

Over the study period, a random sample of 75 eligible women who in the preceding week had attended a 27–28 or 35–36 week gestation antenatal visit were selected each week, using a computerized random number generator. Women were identified using electronic medical records and appointment data, and selected women were mailed an information statement inviting them to participate in a computer-assisted telephone interview (CATI). Non-Aboriginal women were telephoned one-week after the letter was sent, with an online survey offered to those who declined the CATI. In accordance with advice from Aboriginal partners regarding a culturally appropriate recruitment method for Aboriginal women, Aboriginal women and those attending an Aboriginal Maternal Infant Health Service were contacted via text message four days after the letter was sent and invited to complete the survey either online or via CATI. Up to 10 attempts over two weeks were made to contact each woman.

### Control group

Prior to the intervention period, each sector maintained its own usual practice.

### Intervention

#### Model of care

**Fig. 2:**
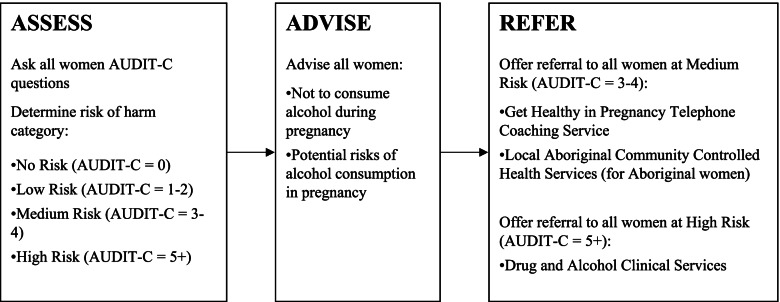
Model of care for addressing alcohol use in pregnancy. AUDIT-C = Alcohol Use Disorders Identification Test-Consumption

A new model of care for addressing alcohol use in pregnancy was developed (Fig. 2). The model of care was based on evidence from systematic reviews [14, 23, 24], and international [12] and national [1] clinical guideline recommendations. It consisted of routine assessment of all women for alcohol consumption in pregnancy using the Alcohol Use Disorders Identification Test-Consumption (AUDIT-C). Based on this assessment, brief advice was provided for women with no/low risk AUDIT-C score (score = 0–2), women with AUDIT-C score = 3–4 (medium risk) were to receive Brief Advice + Referral to a telephone coaching service. High-risk women (AUDIT-C score ≥ 5) were to be provided Brief Advice + offered a referral to Drug and Alcohol Clinical Services [21, 25]. Aboriginal women identified as medium or high risk were offered a choice of referrals to Aboriginal Drug and Alcohol Clinical Services or other self-determined, culturally safe Drug and Alcohol Clinical Services, such as local Aboriginal Community Controlled Health Service. This model of care was to be implemented at the initial antenatal visit (‘Booking in’ visit) and at subsequent antenatal appointments at 28 weeks and 36 weeks [21]. Here, we report the subsequent (28- and 36-week gestation) antenatal appointment data from women surveyed pre- and post-intervention.

#### Implementation strategies

A practice change intervention was implemented over a seven-month period at each of the three participating health sectors to support delivery of the model of care. The intervention consisted of the following seven evidence-based implementation strategies: leadership/managerial supervision [26]; local clinical practice guidelines [27]; an electronic prompt and reminder system [28]; local opinion leaders/champions [26, 29, 30]; educational meetings and materials [31–33]; academic detailing including audit and feedback [34–36]; and monitoring and accountability for the performance of the delivery of healthcare [21, 35]. The development of the practice change intervention has been described in more detail elsewhere [22].

### Data collection

Survey questions were based on previous national surveys and reviewed for cultural appropriateness by Aboriginal women [8, 37, 38]. All interviews were conducted by trained and experienced female interviewers and Aboriginal women were offered the option to undertake the interview with an Aboriginal interviewer. The online survey was built using Research Electronic Data Capture (REDCap) [39], and participants were provided a unique online survey link via email or text message.

### Measures

Self-reported alcohol use was assessed 12 months prior to pregnancy (at their initial antenatal visit – reported for demographic information only); and at subsequent antenatal visits (weeks 28 or 36; defined as alcohol use since the woman “found out [they] were pregnant” – included in analyses pre- and post-intervention).

1. AUDIT-C, scored according to AUDIT-C instructions and Australian alcohol use in pregnancy guidelines cut-points [25, 40]:
  a Total score and component scores
  b Total score classified as No Risk (score = 0) versus Some Risk (score ≥ 1); and Higher risk (score ≥ 3)
2. AUDIT-C components:
  a drinking frequency (“How often would you have a drink containing alcohol?”)
  b drinks per occasion (“How many standard drinks of alcohol would you drink on a typical day when you were drinking?”)
  c ≥ 5 drinks on an occasion (“How often would you have five or more standard drinks on one occasion?”)
3. Special occasion drinking (“Were there any special occasions (e.g., a wedding, anniversary, birthday) since you found out you were pregnant where you consumed any alcohol?”)

Demographic details collected included whether the woman had ever previously been pregnant (first pregnancy: Yes/No), age, socioeconomic disadvantage based on residential postal code (Most disadvantaged included quintiles 1 and 2, and Least disadvantaged included quintiles 4 and 5 from the Index of Relative Socio-Economic Disadvantage (SEIFACAT 2016)) [17]; education level attained; Aboriginal/Torres Strait Islander origin; marital status; employment status; low/high risk antenatal service based on the woman’s antenatal care type (high risk = specialist medical clinics, multi-disciplinary care for women with complex medical needs; low risk = midwifery clinics); and health sector within HNELHD (Greater Newcastle/Peel/Lower Mid-North Coast) [17].

### Statistical analysis

Statistical analysis was conducted using SAS version 9.3. Demographic characteristics were summarized using descriptive statistics. Differences in self-reported alcohol consumption in women attending public maternity services were explored pre-intervention compared to post-intervention. Separate multivariable logistic regression models were used for each of the binary alcohol outcome variables (AUDIT-C score [No risk vs. Some risk]; Special occasion drinking [Yes/No]), controlling for parity, socioeconomic disadvantage, alcohol use pre-pregnancy (AUDIT-C score), education level, age, health sector and month of antenatal visit.

The number of women with AUDIT-C score ≥ 3 (medium to high risk) was so few pre-intervention (*N* = 6) and post-intervention (*N* = 5) that we combined medium and high-risk drinkers, and classified AUDIT-C data as No risk (score = 0) versus Some risk (score ≥ 1) for analysis.

The intervention effects were reported as odds ratios or mean difference with 95% confidence intervals (95%CI), or Pearson’s chi-square statistic, and *p* values.

### Power calculation

With a sample of 1308 and 10.3% at risk of some drinking pre-intervention, a post-intervention sample of 2539 allowed a detectable difference of 3.1% in at risk drinking, with 80% power and an alpha of 0.05.

## Results

**Fig. 3:**
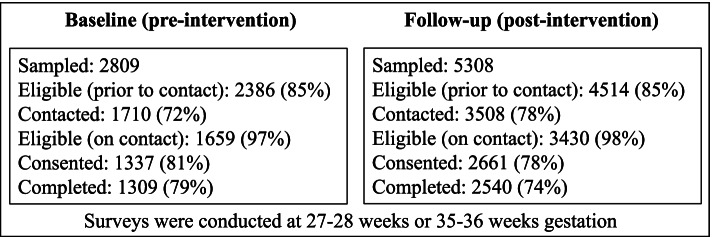
Recruitment flowchart

Figure 3 shows recruitment data for the pre- and post-intervention periods. At pre- and post-intervention, 2809 and 5308 women were selected to participate respectively. Similar proportions were eligible, contacted, consented, and then completed the surveys at the two timepoints (Fig. 3). Surveys were completed by 79% of eligible women pre-intervention (*N* = 1309) and 74% eligible women post-intervention (*N* = 2540).

Demographic details of the women at both time-points are displayed in Table 1 and were similar between groups surveyed pre- and post-intervention. Women were aged approx. 30y, with 40% in their first pregnancy. Most were educated at a tertiary level, married or in a relationship, and employed. According to AUDIT-C assessment, 80.0% and 80.5% women consumed alcohol in the 12 months prior to pregnancy at pre- and post-intervention respectively.

**Table 1:** Demographics of women surveyed before the practice change intervention (pre-intervention) and post-intervention

Characteristic	Pre-intervention (*N* = 1309)	Post-intervention (*N* = 2540)
Age (y)	[*n* = 1308] 29.4 ± 5.3 Median: 29 (18 to 45)	[*n* = 2538] 30.3 ± 5.1 Median: 30 (18 to 51)
First pregnancy	[*n* = 1308] 547 (41.8%)	[*n* = 2538] 1016 (40.0%)
Socioeconomic disadvantage^a^:		[*n* = 2539]
Most disadvantaged	826 (63.1%)	1298 (51.1%)
Least disadvantaged	483 (36.9%)	1241 (48.9%)
Education level:	[*n* = 1307]	[*n* = 2535]
Highschool or less	379 (29.0%)	615 (24.3%)
TAFE certificate or diploma	488 (37.3%)	899 (35.5%)
University, CAE, degree or higher	440 (33.7%)	1021 (40.3%)
Aboriginal/Torres Strait Islander origin (mother)	[*n* = 1308] 80 (6.1%)	[*n* = 2538] 115 (4.5%)
Aboriginal/Torres Strait Islander origin (baby)	[*n* = 1305] 128 (9.8%)	[*n* = 2534] 204 (8.1%)
Marital status:	[*n* = 1307]	[*n* = 2537]
Never married	143 (10.94%)	199 (7.84%)
Married/living together in a relationship	1125 (86.07%)	2289 (90.22%)
Separated/divorced	38 (2.91%)	46 (1.81%)
Widowed	1 (0.08%)	2 (0.08%)
Refused	0	1 (0.04%)
Employment status:	[*n* = 1308]	[*n* = 2538]
Employed full-time	292 (22.32%)	682 (26.87%)
Employed part-time/casual	301 (23.01%)	640 (25.22%)
Unemployed	134 (10.24%)	254 (10.01%)
Can’t work: health reasons	15 (1.15%)	19 (0.75%)
Home duties	236 (18.04%)	339 (13.36%)
Student	41 (3.13%)	55 (2.17%)
Other	10 (0.76%)	10 (0.39%)
On maternity leave: employed full-time prior	144 (11.01%)	300 (11.82%)
On maternity leave: employed part-time/casual prior	135 (10.32%)	239 (9.42%)
Antenatal service^b^:		[*n* = 2531]
High risk	708 (54.09%)	1302 (51.44%)
Low risk	581 (44.39%)	1190 (47.02%)
AMIHS	20 (1.53%)	39 (1.54%)
Health sector:		
Greater Newcastle (urban)	860 (65.70%)	2180 (85.83%)
Peel (regional/rural)	201 (15.36%)	268 (10.55%)
Lower Mid-North Coast (regional/ rural)	248 (18.95%)	92 (3.62%)
AUDIT-C score in 12 months before pregnancy (median (range))	[*n* = 1298] 2 (0 to 12)	[*n* = 2518] 3 (0 to 12)

*AMIHS* Aboriginal Maternal and Infant Health Service, *AUDIT-C* Alcohol Use Disorders Identification Test-Consumption

^a^Socioeconomic disadvantage was classified using the Index of Relative Socio-Economic Disadvantage (SEIFACAT 2016). Most disadvantaged included quintiles 1 and 2, and Least disadvantaged included quintiles 4 and 5

^b^High risk antenatal services included medical clinic, women with vulnerabilities, and women with complex medical needs; Low risk antenatal services were midwifery clinics

### Difference in alcohol use

Due to small numbers, and failure to satisfy the requirement of independence of observations for Mann–Whitney U tests, intervention effects were not explored for all alcohol use outcomes including: drinking frequency, number of drinks per occasion, and frequency of consuming ≥ 5 drinks on one occasion. The median AUDIT-C score pre- and post-intervention was 0 (Table 2). There was no statistically significant effect of the intervention on the proportion of women in each AUDIT-C risk category (No risk versus any risk; *p* = 0.08). However, a significant reduction was observed in the proportion with special occasion drinking (Pre-intervention: 11.59%; Post-intervention: 8.43%; OR: 0.60 (95%CI: 0.46 to 0.79); *p* < 0.001) (Table 2).

**Table 2:** Alcohol use during subsequent antenatal visits before and after implementation of the practice change intervention

Alcohol use during pregnancy measure	Pre-intervention (*n* = 1308)	Post-intervention (*n* = 2539)	Mean difference, OR (95%CI), or X^2^	*p*
AUDIT-C score:				
Median (range)	0 (0 to 7)	0 (0 to 5)	-	-
AUDIT-C score^a^:			No vs Some risk:	0.08
No risk (0) (N (%))	1173 (89.68)	2304 (90.74)	0.80 (0.62, 1.03)	
Some risk (≥ 1) (N (%))	135 (10.32)	235 (9.26)		
Higher risk (≥ 3) (N (%))	6 (0.46)	5 (0.20)		
Drinking frequency		[*n* = 2538]	-	-
Never (N (%))	1173 (89.68)	2303 (90.71)		
Monthly or less (N (%))	111 (8.49)	202 (7.96)		
2-4x/month (N (%))	19 (1.45)	33 (1.30)		
2-3x/week (N (%))	4 (0.31)	0		
≥ 4x/week (N (%))	1 (0.08)	0		
N drinks per occasion:	[*n* = 135]	[*n* = 235]	-	-
1–2 (N (%))	133 (98.52)	230 (97.87)		
3–4 (N (%))	1 (0.74)	2 (0.85)		
5–6 (N (%))	1 (0.74)	2 (0.85)		
7–9 (N (%))	0	1 (0.43)		
≥ 10 (N (%))	0	0		
Frequency of ≥ 5 drinks on 1 occasion:	[*n* = 135]	[*n* = 235]	-	-
Never (N (%))	132 (97.78)	230 (97.87)		
< Monthly (N (%))	1 (0.74)	5 (2.13)		
Monthly (N (%))	2 (1.48)	0		
Weekly (N (%))	0	0		
Daily or almost daily (N (%))	0	0		
Special occasion drinking^1^ (N (%))	[*n* = 1087] 126 (11.59)	214 (8.43)	OR: 0.60 (0.46 to 0.79)	< 0.001*

*95%CI* 95% confidence interval, *AUDIT-C* Alcohol Use Disorders Identification Test-Consumption, *OR* odds ratio

^a^Logistic regression models were adjusted for parity, disadvantage, AUDIT-C score pre-pregnancy, education, health sector, month of appointment, and age

## Discussion

This study was the first to explore whether a practice change intervention that resulted in improvements in routine antenatal care and screening translated into reductions in alcohol consumption for pregnant women. A statistically significant improvement was observed in self-reported special occasion drinking but not in overall risk level of alcohol consumption (AUDIT-C).

The design of the model of care was informed by international and national antenatal clinical practice guidelines [21], and systematic review evidence on effective interventions for increasing abstinence during pregnancy [23]. A formative cross-sectional survey of antenatal clinicians and managers within the participating public maternity services on barriers to care provision informed the practice change strategies [41]. Although the practice change intervention significantly improved women’s reported receipt of advice and care [22] post-intervention rates of care provision remained low. This suggests that barriers to implementation of the model of care into routine practice persisted [22, 41]. Efforts to refine the implementation strategies are needed to ensure all women receive evidence-based care. This should include reassessment of priority barriers and facilitators of care delivery and development of additional implementation strategies based on effective behaviour change techniques [42].

However, despite low numbers receiving the intervention intended, there was a significant reduction in the proportion of women reporting special occasion drinking after the intervention. It is possible that special occasion drinking behaviour is easier to change because it is not habitual like regular drinking. Special occasion drinking may be considered infrequent behaviour, and in contrast, regular drinking may be considered ‘habitual’, and more resistant to change despite knowledge of the potential harms [43]. Our finding reinforces the importance of asking pregnant women about special occasion drinking, in line with recommendations by Muggli et al., who reported high rates of binge level special occasion drinking in pregnant women [8]. In the present cohort, 33.3% women who reported drinking during pregnancy only consumed alcohol on special occasions, mostly (91.3%) at low levels (1–2 drinks per occasion) [10]. This indicates that special occasion drinking should be defined not only as binge level drinking, but as any amount of alcohol during special occasions, to maximise detection of risk to the unborn child. This is an important outcome because any amount of alcohol has the potential to harm the unborn child and no safe lower limit has been established.

One possible reason why we did not observe any significant change in the proportion of women who reported “regular” alcohol use during pregnancy (AUDIT-C) is that information alone does not change behaviour. Previous research by our group [10, 44, 45] and others [20, 46, 47] suggests that attitudes to alcohol use in pregnancy are more important determinants of maternal behaviour than knowledge of alcohol harms. Additionally, although we observed a significant increase in health providers who implemented all recommended components of the intervention in clinical practice, there is still potential for increasing the proportion of women receiving the recommended care. Although we did not measure attitudinal change in this trial, we previously reported in this cohort that a positive attitude towards alcohol use in pregnancy was predictive of ongoing alcohol use in pregnancy [10].

The model of care to address alcohol use in pregnancy focused on clinical care, and did not address social aspects of alcohol use. Women’s ability to make positive life changes to protect their baby from harm is mediated by social and structural determinants of their health and wellbeing, including peer drinking, social relationships and norms, stigma, trauma and other stressors [48]. Assessment of and support for these contributors to ongoing drinking, in addition to implementation of a standard model of antenatal care based on guideline recommendations, may prove more effective in reducing alcohol use during pregnancy than addressing any one factor alone. Although the standard model of antenatal care currently includes referral to services that address these social aspects of alcohol use (e.g., Drug and Alcohol Services), the observed rate of referral was low [22]. To ensure women have access to the most effective care, it is important to increase clinician’s offer of referral for women consuming alcohol in pregnancy at medium to high risk levels as well as women’s uptake of these services.

This study had many strengths. It was a multi-site randomized trial with a stepped-wedge design, tested in public antenatal services in rural and urban sites with a large sample of pregnant women. We also used two different outcome measures to assess alcohol consumption (AUDIT-C and special occasion drinking). A number of potential limitations need to be mentioned. In this study we report secondary outcomes that were not specifically powered in the study design. Although the study was adequately powered to detect differences of 3.1%, based on a pre-intervention prevalence of ‘at risk’ alcohol use (AUDIT C score ≥ 1) during pregnancy of 10.3%, the intervention did not significantly reduce the proportion of women reporting regular drinking. Additionally, although we controlled for potential confounders and our study was adequately powered, we cannot be certain that the reduction in special occasion drinking was a result of the intervention.

## Conclusions

Our results show that the implementation of a practice change intervention to support the introduction of a model of evidence-based care does reduce special occasion drinking, but not other patterns of alcohol use in pregnancy. The barriers to implementing the model of care in antenatal service settings, as well as barriers experienced by individual women to changing their alcohol use behaviour, must be identified and addressed so that all women receive evidence-based care and are supported to abstain from alcohol consumption during pregnancy.

## Data Availability

The datasets used and/or analyzed during the current study are available from the corresponding author on reasonable request.

## Abbreviations

95%CI: 95% Confidence interval
AMIHS: Aboriginal Maternal and Infant Health Services
AUDIT-C: Alcohol Use Disorders Identification Test-Consumption
CATI: Computer-assisted telephone interview
FASD: Fetal alcohol spectrum disorder
HNELHD: Hunter New England Local Health District
NHMRC: National Health and Medical Research Council
NSW: New South Wales
OR: Odds ratio
PAE: Prenatal alcohol exposure
REDCap: Research Electronic Data Capture
SEIFACAT: Socio-economic indexes for areas categories
Wks: Weeks

## References

[1] National Health and Medical Research Council, Australian Research Council and Universities Australia (2020). Australian guidelines to reduce health risks from drinking alcohol.

[2] Oei JL (2020). Alcohol use in pregnancy and its impact on the mother and child. Addiction.

[3] International Alliance for Responsible Drinking. Drinking guidelines for pregnancy and breastfeeding 2019 [Available from: http://iardwebprod.azurewebsites.net/science-resources/detail/Drinking-Guidelines-for-Pregnancy-and-Breastfeedin.

[4] Williams L. UK Chief Medical Officers' Low Risk Drinking Guidelines 2016 [Available from: https://assets.publishing.service.gov.uk/government/uploads/system/uploads/attachment_data/file/545937/UK_CMOs__report.pdf.

[5] Butt P, Beirness D, Gliksman L, Paradis C, Stockwell T. Alcohol and health in Canada: A summary of evidence and guidelines for low risk drinking. Ottawa: Canadian Centre on Substance Abuse; 2011.

[6] Popova S, Lange S, Probst C, Gmel G, Rehm J. Estimation of national, regional, and global prevalence of alcohol use during pregnancy and fetal alcohol syndrome: a systematic review and meta-analysis. Lancet Glob Health. 2017 10.1016/S2214-109X(17)30021-9. 10.1016/S2214-109X(17)30021-9 28089487

[7] Anderson AE, Hure AJ, Forder P, Powers JR, Kay-Lambkin FJ, Loxton DJ (2013). Predictors of antenatal alcohol use among Australian women: a prospective cohort study. BJOG.

[8] Muggli E, O'Leary C, Donath S, Orsini F, Forster D, Anderson PJ (2016). "Did you ever drink more?" a detailed description of pregnant women's drinking patterns. BMC Public Health.

[9] McCormack C, Hutchinson D, Burns L, Wilson J, Elliott E, Allsop S (2017). Prenatal alcohol consumption between conception and recognition of pregnancy. Alcohol Clin Exp Res.

[10] Tsang TW, Kingsland M, Doherty D, Anderson AE, Tully B, Crooks K (2022). Predictors of alcohol use during pregnancy in Australian women. Drug Alcohol Rev.

[11] McBride N, Carruthers S, Hutchinson D (2012). Reducing alcohol use during pregnancy: listening to women who drink as an intervention starting point. Glob Health Promot.

[12] World Health Organization (2014). Guidelines for identification and management of substance use and substance use disorders in pregnancy.

[13] Department of Health (2020). Clinical Practice Guidelines.

[14] Ujhelyi Gomez K, Goodwin L, Jackson L, Jones A, Chisholm A, Rose AK (2021). Are psychosocial interventions effective in reducing alcohol consumption during pregnancy and motherhood? A systematic review and meta-analysis. Addiction.

[15] Wangberg SC (2015). Norwegian midwives' use of screening for and brief interventions on alcohol use in pregnancy. Sex Reprod Healthc.

[16] Kesmodel U, Schiøler KP (2002). Drinking during pregnancy: Attitudes and knowledge among pregnant Danish women, 1998. Alcohol Clin Exp Res.

[17] Doherty E, Wiggers J, Wolfenden L, Anderson AE, Crooks K, Tsang TW (2019). Antenatal care for alcohol consumption during pregnancy: pregnant women’s reported receipt of care and associated characteristics. BMC Pregnancy Childbirth.

[18] Kesmodel US, Kesmodel PS (2011). Alcohol in pregnancy: attitudes, knowledge, and information practice among midwives in Denmark 2000 to 2009. Alcohol Clin Exp Res.

[19] Waller A, Bryant J, Cameron E, Galal M, Quay J, Sanson-Fisher R (2016). Women’s perceptions of antenatal care: are we following guideline recommended care?. BMC Pregnancy Childbirth.

[20] Bazzo S, Battistella G, Riscica P, Moino G, Marini F, Bottarel M (2015). Evaluation of a multilevel and integrated program to raise awareness of the harmful effects of prenatal alcohol exposure in a local community. Alcohol Alcohol.

[21] Kingsland M, Doherty E, Anderson AE, Crooks K, Tully B, Tremain D (2018). A practice change intervention to improve antenatal care addressing alcohol consumption by women during pregnancy: research protocol for a randomised stepped-wedge cluster trial. Implement Sci.

[22] Doherty D, Kingsland M, Elliott EJ, Tully B, Wolfenden L, Dunlop A (2022). Practice change intervention to improve antenatal care addressing alcohol consumption during pregnancy: a randomised stepped-wedge controlled trial. BMC Pregnancy Childbirth..

[23] Stade BC, Bailey C, Dzendoletas D, Sgro M, Dowswell T, Bennett D. Psychological and/or educational interventions for reducing alcohol consumption in pregnant women and women planning pregnancy. The Cochrane database of systematic reviews. 2009(2):Cd004228. 10.1002/14651858.CD004228.pub2 PMC4164939 19370597

[24] Nilsen P (2009). Brief alcohol intervention to prevent drinking during pregnancy: an overview of research findings. Curr Opin Obstet Gynecol.

[25] Foundation for Alcohol Research and Education (FARE). Information for health professionals on assessing alcohol consumption in pregnancy using AUDIT-C. Australian Government Department of Health; 2017.

[26] Flodgren G, Parmelli E, Doumit G, Gattellari M, O'Brien MA, Grimshaw J, et al. Local opinion leaders: effects on professional practice and health care outcomes. The Cochrane database of systematic reviews. 2011(8):Cd000125. 10.1002/14651858.CD000125.pub4 PMC4172331 21833939

[27] Rotter T, Kinsman L, James E, Machotta A, Gothe H, Willis J, et al. Clinical pathways: effects on professional practice, patient outcomes, length of stay and hospital costs. The Cochrane database of systematic reviews. 2010(3):Cd006632. 10.1002/14651858.CD006632.pub2 20238347

[28] Shojania KG, Jennings A, Mayhew A, Ramsay CR, Eccles MP, Grimshaw J (2009). The effects of on-screen, point of care computer reminders on processes and outcomes of care. Cochrane Database Syst Rev.

[29] Woo K, Milworm G, Dowding D (2017). Characteristics of quality improvement champions in nursing homes: a systematic review with implications for evidence-based practice. Worldview Evid-Based Nurs.

[30] Welsh SM, Sherriff A, Flodgren G (2019). The champion for improved delivery of care to older people in long-term care settings: effects on professional practice, quality of care and resident outcomes. Cochrane Database Syst Rev.

[31] Dray J, Licata M, Doherty E, Tully B, Williams B, Curtin S (2022). Enhancing clinician participation in quality improvement training: implementation and impact of an evidence-based initiative to maximise antenatal clinician participation in training regarding women's alcohol consumption during pregnancy. BMC Health Serv Res.

[32] Reeves S, Perrier L, Goldman J, Freeth D, Zwarenstein M (2013). Interprofessional education: effects on professional practice and healthcare outcomes (update). Cochrane Database Syst Rev.

[33] Forsetlund L, Bjørndal A, Rashidian A, Jamtvedt G, O'Brien MA, Wolf F (2009). Continuing education meetings and workshops: effects on professional practice and health care outcomes. Cochrane Database Syst Rev.

[34] Chaillet N, Dubé E, Dugas M, Audibert F, Tourigny C, Fraser WD (2006). Evidence-based strategies for implementing guidelines in obstetrics: a systematic review. Obstet Gynecol.

[35] Ivers N, Jamtvedt G, Flottorp S, Young JM, Odgaard-Jensen J, French SD, et al. Audit and feedback: effects on professional practice and healthcare outcomes. The Cochrane database of systematic reviews. 2012(6):Cd000259. 10.1002/14651858.CD000259.pub3 PMC11338587 22696318

[36] O'Brien MA, Rogers S, Jamtvedt G, Oxman AD, Odgaard-Jensen J, Kristoffersen DT (2007). Educational outreach visits: effects on professional practice and health care outcomes. Cochrane Database Syst Rev.

[37] Australian Institute of Health and Welfare (AIHW) (2020). National Drug Strategy Household Survey 2019. Drug Statistics series no. 32. PHE 270.

[38] Hutchinson D, Wilson J, Allsop S, Elliott E, Najman J, Burns L (2018). Cohort Profile: the triple B pregnancy cohort study: a longitudinal study of the relationship between alcohol, tobacco and other substance use during pregnancy and the health and well-being of Australian children and families. Int J Epidemiol.

[39] Harris PA, Taylor R, Thielke R, Payne J, Gonzalez N, Conde JG (2009). Research electronic data capture (REDCap)–a metadata-driven methodology and workflow process for providing translational research informatics support. J Biomed Inform.

[40] Bush K, Kivlahan D, McDonell M, Fihn S, Bradley K (1998). The AUDIT alcohol consumption questions (AUDIT-C): an effective brief screening test for problem drinking. ambulatory care quality improvement project (ACQUIP). alcohol use disorders identification test. Arch Intern Med..

[41] Doherty E, Kingsland M, Wiggers J, Anderson AE, Elliott EJ, Symonds I (2019). Barriers to the implementation of clinical guidelines for maternal alcohol consumption in antenatal services: A survey using the theoretical domains framework. Health Promot J Austr.

[42] Chauhan BF, Jeyaraman M, Mann AS, Lys J, Skidmore B, Sibley KM (2017). Behavior change interventions and policies influencing primary healthcare professionals’ practice—an overview of reviews. Implementation Sci.

[43] Barker JM, Taylor JR (2014). Habitual alcohol seeking: modeling the transition from casual drinking to addiction. Neurosci Biobehav Rev.

[44] Peadon E, Payne J, Henley N, D'Antoine H, Bartu A, O'Leary C (2010). Women's knowledge and attitudes regarding alcohol consumption in pregnancy: a national survey. BMC Public Health.

[45] Peadon E, Payne J, Henley N, D'Antoine H, Bartu A, O'Leary C (2011). Attitudes and behaviour predict women's intention to drink alcohol during pregnancy: the challenge for health professionals. BMC Public Health.

[46] Raymond N, Beer C, Glazebrook C, Sayal K (2009). Pregnant women's attitudes towards alcohol consumption. BMC Public Health.

[47] Watt MH, Eaton LA, Dennis AC, Choi KW, Kalichman SC, Skinner D (2016). Alcohol use during pregnancy in a South African community: Reconciling knowledge, norms, and personal experience. Matern Child Health J.

[48] Lyall V, Wolfson L, Reid N, Poole N, Moritz KM, Egert S (2021). “The problem is that we hear a bit of everything…”: A qualitative systematic review of factors associated with alcohol use, reduction, and abstinence in pregnancy. International journal of environmental research and public health..

